# Change in the Low-Cycle Performance on the 3D-Printed Materials ABS, ASA, HIPS, and PLA Exposed to Mineral Oil

**DOI:** 10.3390/polym16081120

**Published:** 2024-04-17

**Authors:** Marcin Głowacki, Adam Mazurkiewicz, Katarzyna Skórczewska, José Miguel Martínez Valle, Emil Smyk

**Affiliations:** 1 Faculty of Mechanical Engineering, Bydgoszcz University of Science and Technology, Kaliskiego 7 Street, 85-796 Bydgoszcz, Poland; adam.mazurkiewicz@pbs.edu.pl (A.M.); emil.smyk@pbs.edu.pl (E.S.); 2 Faculty of Chemical Technology and Engineering, Bydgoszcz University of Science and Technology, Seminaryjna 3 Street, 85-326 Bydgoszcz, Poland; katarzyna.skorczewska@pbs.edu.pl; 3 Department of Mechanics, Building Leonardo Da Vinci, Campus of Rabanales, University of Córdoba, Cta. Madrid-Cádiz, Km. 396, 14071 Córdoba, Spain; jmvalle@uco.es

**Keywords:** cyclic test, mineral oil, polymers, 3D print, micro-CT, oil bath

## Abstract

Three-dimensionally printed parts are increasingly used in industry for quick repairs. They are often operated in the presence of grease, oil, and others. This article describes the effect of engine mineral oil on the fatigue life of 3D-printed FDM plastic samples. For this reason, this article aimed to investigate the influence of oil on the fatigue life of materials made using this technology. Samples made of ABA, ASA, PLA, and HIPS materials were printed with 100% fill. Divided into groups, they were stored for 15, 30, and 60 days in an oil bath at a room temperature of 23 °C and an increased temperature of 70 °C. To compare the effect of storage in oil, static tests were performed to determine the tensile strength of the specimens and to determine the load levels for the cyclic tests. Cyclic tests were performed to determine the effect of oil and temperature on the fatigue life. Internal structure studies of the specimens were performed using computed microtomography to determine the changes in the porosity of the specimens under the influence of oil. In the case of ABS, the oil-bathed samples showed a clear increase in the fatigue life, especially at 23 °C. For the ASA specimens, an increase was also evident, especially for the lower stress value. For HIPS and PLA, no clear effect of the oil bath on the fatigue life value of the samples was determined. Porosity studies using computed microtomography showed a clear decrease in the porosity of the samples as a result of the oil bath for all of them.

## 1. Introduction

Currently, additive manufacturing technologies are identified as promising areas in the production of machine elements, parts, and components due to the reduction in production costs, waste, or availability constraints [1]. The most common 3D printing technology is Fused Deposition Modeling (FDM). In this technology, thermoplastic materials are used in the manufacturing process. The thermoplastic materials utilized in 3D printing include ABS (acrylonitrile–butadiene–styrene), which is deemed a universal material and is employed in various industries such as toy manufacturing; ASA (acrylonitrile–styrene–acrylate), due to its UV resistance, is used in the automotive industry and the production of bumper covers or side mirror housings; HIPS (high-impact polystyrene), due to its high impact strength, is used in the production of helmets and protective elements; PLA (poly(lactic acid)), as a biodegradable material, is suitable for the production of disposable vessels; PETG (polyethylene terephthalate glycol) is a material from which plastic bottles are made; PC (polycarbonate), due to its high mechanical resistance, is frequently encountered in the automotive industry; TPU (thermoplastic polyurethane), as a flexible material, can be a material for the production of toys and elements that cannot be brittle. Many materials are not suitable for 3D printing, including liquid materials, pastes, silicones, and photopolymer resins; usually, a different technology is used for these materials [2,3,4,5]. Knowledge of the environmental and site-dependent impacts of 3D-printed products contributes to an increase in the possible service life. New materials and their modifications are being developed that increasingly meet user expectations in terms of specified properties [6,7,8,9]. As a result, the range of applications of plastics is also expanding, and they are increasingly used, for example, for parts such as gears or the bodies and covers of mechanisms. As a result, plastic parts operating in this capacity are exposed to environmental factors such as engine oil, mineral oil, and lubricating agents [10]. There are also other applications, primarily in the automotive industry, where 3D printing is used to produce lightweight spare parts and prototypes [11]; in the electronics industry, where cases and packaging are designed as people’s interest turns toward wearable electronics [12]; in the furniture industry, where spare parts and various accessories are produced; as well as in the food industry, where packaging and storage containers are produced [13]. Engine oil is one of the most common substances found in the operating environment of machinery and equipment. There are three types of oil available on the market: synthetic oil, semi-synthetic oil, and mineral oil. The difference between these types of oil is related to the level of viscosity [14]. Oil is used for lubrication, that is, to reduce the frictional resistance of machine components moving against each other, and to cool them, which increases their lifespan. Depending on the needs, it can contain various additives, which can have a negative impact on the durability of the components through the possible migration, swelling, or change in their desired characteristics, such as mechanical and thermal properties [15].

This paper describes the effect of mineral oil on the durability of the following four commonly used plastics for 3D printing: ABS, ASA, HIPS, which belongs to the group of styrene materials, and PLA, which belongs to the group of biopolymers and is biodegradable, which is an important feature nowadays [16,17]. These materials were also selected because they are among the most frequently used in FMA technology production [18]. They are widely available and often used by small- and medium-sized enterprises.

Most typical structural components are usually subject to time-varying loads during operation. The phenomenon of material deterioration due to such loads is called fatigue. These loads are generally lower than the static strength of the material. A large number of cycles of such loading causes non-reversible damage before reaching its maximum strength [19]. 

The most commonly described studies of the mechanical properties for 3D-printed parts focus on evaluating the relationship of the printing parameters to the mechanical properties of the printed samples [13]. The influence of the nozzle diameter, sample placement on the table, layer thickness, fill type, or raster width is evaluated [20]. There are also articles in which cyclic tests were used to evaluate the strength. Articles [21,22] focused on PLA material and its modified counterpart, where it was shown that, under the same loading conditions, the fatigue life of the 3D-printed samples was similar. Additionally, the presence of porosity in the material was demonstrated by conducting SEM (scanning electron microscope) analyses [23,24]. Another paper performed a low-cycle fatigue strength analysis for TPU (thermoplastic polyurethane). The material, in the form of pellets, along with paraffin, was extruded into filament form to be able to print test shapes using a 3D printer. The results showed a reduction in the fatigue life [25]. The applications of the described materials varied from use in sports equipment, shoe insoles for runners, or designing scaffolds that can replace human bone. Among the studies mentioned, most were concerned with evaluating the low-cycle fatigue strength of specimens that were tested without the influence of environmental factors on the structure. So far, there is little or no information reported in the literature presenting the effect of environmental factors on the mechanical properties determined in low-cycle tests. This raises the question of how the number of cycles can be affected by factors in the industrial environment, e.g., by exposing the material to mineral/engine oil [26]. The results of the research may result in a better representation of the various real mechanical behaviors of the materials during cyclic loading [27]. Such studies can also give information about the behavior of the invariability of the performance properties of the parts made by 3D printing from polymers in specific operating environments [28].

In the present study, 3D-fabricated samples of commonly used 3D printing materials such as ABS, ASA, HIPS, and PLA were placed in machine oil for up to 60 days at 23 °C and 70 °C. Mineral oil was selected because of its widespread use as a lubricant and cooling agent in many industrial applications. The purpose of this study is to determine how environmental conditions in the form of the exposure of fittings to mineral oil affect the mechanical properties in low-cycle fatigue cyclic tests of polymeric materials for 3D printing. Such an evaluation will allow for a more precise selection of materials for specific applications. This, in turn, could lead to more efficient, durable, and safety-free products and structures incorporating 3D-printed parts. 

This study determined the differences in cyclic strength by measuring the number of load cycles performed to specimen failure and evaluated the changes in the specimen porosity to determine the effects of mineral oil and temperature on the mechanical properties of 3D-printed parts operating in contact with oil.

## 2. Materials and Methods

### 2.1. Materials and Printing Procedures

All of the printing materials that were used during the study were from the Polish manufacturer Spectrum (PL) according to the name available on the manufacturer’s website, including the Smart ABS, ASA 275, HIPS-X, and PLA Premium [29]. A mineral oil labeled “Platinum Classic” produced by Orlen (PL) was used in the experiment. This oil is characterized by the specification 15W-40. A Zortrax m200 plus printer (Zortrax S.A., Olsztyn, Poland) was used for printing. The default printing parameters recommended by the printer’s manufacturer in the ZSuit software (https://zortrax.com/software/) supplied with the printer were used to produce the samples. The software offers various possibilities for the printing process for each material, such as nozzle temperature, printing speed, layer thickness, layer height, and many others. In our case, the only modification made to the default parameters was to set the fill to 100% and select a linear pattern, in accordance with the sample fabrication methodology included in our earlier paper [28]. A fill of 100% should enable it not to penetrate the inner layers of the structure and, despite the higher material consumption, increase the strength of the fabricated parts. The linear infill pattern allows the material to be applied in parallel lines, which should lead to a higher tensile strength along the infill axis [20,21]. 

**Figure 1:**
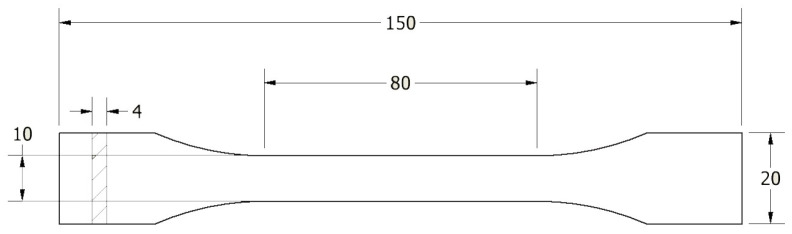
Shape and dimensions of the sample used for testing according to EN ISO 527-1:2012 [30].

The specimens were designed according to EN ISO 527-1:2012 [30], which describes how to determine mechanical properties in static tension and the shape and dimensions of the specimens used for such tests. The shape and dimensions of the specimen used in this study are shown in Figure 1.

### 2.2. Preparation of Samples

Samples of materials (ABS, ASA, HIPS, and PLA) were divided into the following 4 groups:

- G0—a group of reference samples not treated with oil;
- G15—a group of samples sub-treated with oil for 15 days;
- G30—a group of samples treated with oil for 30 days;
- G60—a group of samples treated with oil for 60 days.

As mentioned, the printing parameters of the sample manufacturing were the same as in [28]. Samples from the G15, G30, and G60 groups were divided into two subgroups that were exposed to oil at 23 °C and 70 °C. Samples intended for testing at 70 °C, after being placed in machine oil, were placed in an industrial dryer to maintain the temperature, while the rest of the samples immersed in oil were placed in an area not exposed to light at a room temperature of 23 °C. After the storage period, the samples were removed from the oil, the excess of which was removed with a cloth in preparation for the mechanical testing. The reference group for G15, G30, and G60 was G0, which was not exposed to oil and elevated temperature. 

### 2.3. Static Test

Static tensile testing was carried out for the specimens from the G0 control group on an Instron E3000 testing machine (Instron, Canton, MA, USA) in accordance with the standard for plastics, EN ISO 527-1:2012 [30]. Based on the values of maximum stress, *σ_m_*, obtained from the test, the load levels in the cyclic tests were determined for the specimens in the other groups. The tensile strength values and load levels for the other plastics are shown in Table 1. 

### 2.4. Cyclic Test

Cycle tests were conducted for the specimens from all groups. An Instron E3000 testing machine was used to evaluate the low-cycle fatigue strength of the specimens. The specimens were subjected to forces from the zero pulses, and the value of the amplitude stress, σ_a_, was related in percentage to the tensile strength, as described in Section 2.3. At each load level, 3 specimens were tested in series [31]. Tests were conducted at a frequency of 2 Hz, and the number of load cycles to specimen failure was recorded.

### 2.5. Porosity Measurement

To evaluate changes in the internal structure of the samples after exposure to an environmental agent in the form of an oil bath, X-ray microtomography of the samples (micro-CT) was performed. The study was carried out on a vivaCT 80 device from Scanco (Scanco. A.G., Brüttisellen, Switzerland). The scanning parameters were as follows: 55 kVp, 145 mA, a filter Alu of 0.5 mm, an integration time of 200 ms, and a resolution of 24 µm. The scanning time per sample was 66 min. During the test, changes in the porosity of the samples from each group were evaluated in comparison with the control group. The comparison was carried out at a length of 10 mm in the middle of the operating part of each sample before the mechanical testing. The number of samples tested by the group is shown in Table 2.

### 2.6. Statistics

Origin 8.6 Pro software with implemented statistical analysis modules was used for the statistical analysis of the obtained results. ANOVA with the post hoc Tukey test was used to compare the significant differences for each mean value. The normal distribution was confirmed using the Shapiro–Wilk test and the homogeneity of variance was confirmed using Levene’s test. All analyses were performed assuming a significance level below 0.05. A similar method was used in our previous publication [31].

## 3. Results

### 3.1. Cyclic Test Results

**Figure 2:**
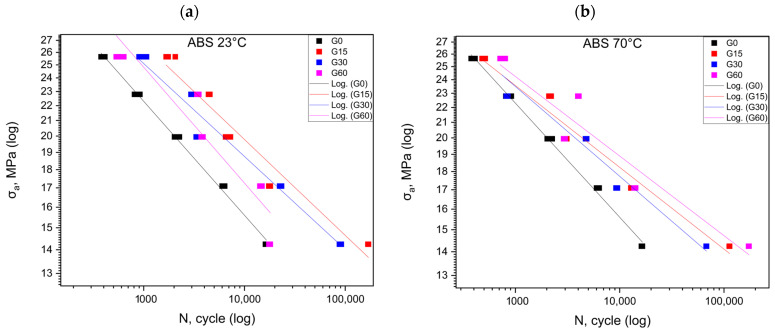
Fatigue curves for ABS at 23 °C (**a**) and at 70 °C (**b**).

**Figure 3:**
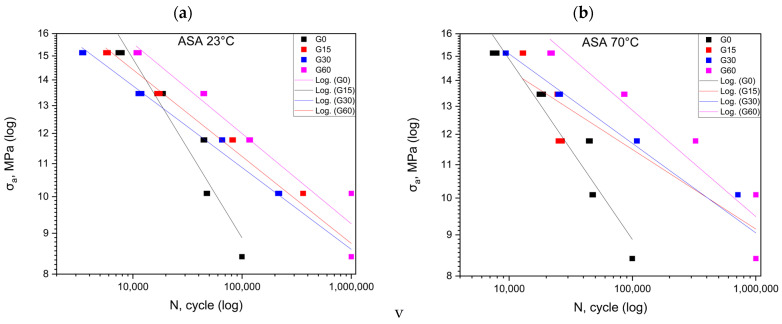
Fatigue curves for ASA at 23 °C (**a**) and at 70 °C (**b**).

**Figure 4:**
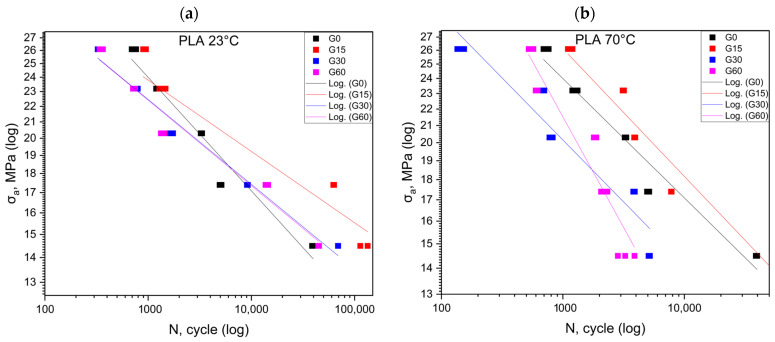
Fatigue curves for PLA at 23 °C (**a**) and at 70 °C (**b**).

**Figure 5:**
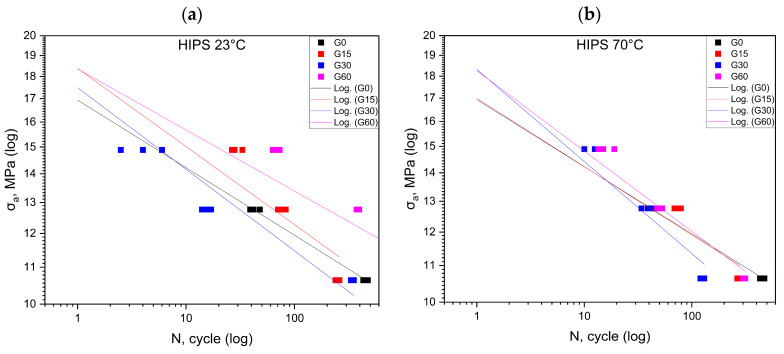
Fatigue curves for HIPS at 23 °C (**a**) and at 70 °C (**b**).

The graphs created from the fatigue test results are summarized in Figure 2, Figure 3, Figure 4 and Figure 5. Figure 2 shows the results for the ABS material at 23 °C and 70 °C. The analysis of the results indicates an observable upward trend for the first cycle conducted at room temperature (Table 3). These values undergo a reduction of almost 50% in the second cycle, and after the third cycle, the results are almost identical to the reference data. In the context of experiments conducted at elevated temperatures, we observe the increased durability of the samples after each cycle. Among all the results, the highest number of cycles were withstood by the samples subjected to the third cycle.

Figure 3 shows the results for the ASA material at 23 °C and 70 °C. We observe increased durability for the fatigue cycles, both at room temperature and the elevated temperature, from the first cycle onwards (Table 4). This value exhibited an upward trend with the longer exposure time of the samples to the mineral oil. Samples that were subjected to the action of elevated temperature demonstrated greater durability for the fatigue cycles. These values at individual levels were multiple times higher compared to those obtained at room temperature.

Figure 4 shows the results for the PLA material at 23 °C and 70 °C. The greatest durability for the fatigue cycles is observed for the first cycle conducted at room temperature (Table 5). This durability decreases with the longer exposure time of the samples to the mineral oil. In the case of the experiments conducted at a temperature of 70 degrees Celsius, we observe a slight increase in the durability for the first cycle. However, this durability drastically falls below the level of the reference test as the exposure time to the mineral oil is extended.

Figure 5 shows the results for the HIPS material at 23 °C and 70 °C. Conducting cyclic fatigue tests at high stress levels is not feasible for the material designated as HIPS (Table 6). The greatest increase in durability was recorded in the third cycle conducted at room temperature. In the case of the experiments conducted at elevated temperatures, as well as for the remaining results, the durability was below the values obtained in the reference test.

Table 7 summarizes the equations of the fatigue curves that are shown in Figure 1, Figure 2, Figure 3 and Figure 4. The coefficient of determination R^2^ was higher than 0.87, which means a high level of correlation. The equations of the fatigue curves were not present in Figure 2, Figure 3, Figure 4 and Figure 5 so as not to reduce the readability of the charts.

### 3.2. Statistical Analysis of Cyclic Test Results

Figure 2, Figure 3, Figure 4 and Figure 5 made in the log(σ_a_)—log(N) coordinates (strain number of cycles to damage sample) plotted points corresponding to the results obtained from each sample. These points were described by a regression line. To check whether the regression line well described the obtained results, Student’s *t* test was performed at the assumed significance level of *p* = 0.05. The test results in all cases showed the correctness of the adopted regression curves. In addition, the normality of the distribution of the residuals was checked with the Shapiro–Wilk test. The results of the test indicated that there were no grounds for questioning the normality of the distribution.

To calculate statistical differences between the studied groups for each material, a test of the equality of the directional coefficients and free expressions of the regression equations writing the experimental results was performed. The results of the test confirmed that the regression curves describing the results obtained for each group were statistically different

### 3.3. Changes in the Porosity of Samples

**Figure 6:**
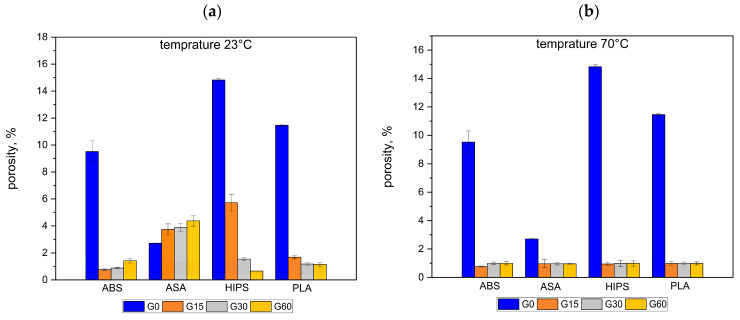
Porosity for different storage times in oil at temperatures of 23 °C (**a**) and 70 °C (**b**).

The porosity change values of the samples are shown in Figure 6. Figure 6a shows the changes in the average porosity values of the samples stored in oil for 15, 30, and 60 days at 23 °C. Figure 6b shows the porosity changes for the samples stored at 70 °C. The exact numerical values are shown in Table 8 and Table 9.

The porosity changes had a different course depending on the type of plastic at a temperature of 23 °C. For ABS, the initial decrease in the porosity for the G15 group was followed by an increase for the G30 and G60 groups. For ASA, an increase in the porosity was observed in groups G15, G30, and G60 concerning the reference group. The increases were greater the longer the samples were stored in oil. For HIPS and PLA, there was a decrease in the porosity the longer the time that the samples were stored in oil. 

The high porosity of the samples from the G0 groups, especially those made from PLA and HIPS, was more than 10%. As mentioned earlier, the prints were made with 100% fill. Such a high porosity may indicate that the manufacturer’s recommended printing parameters for these plastics are not optimal.

Changes in the porosity of the samples of all groups were evaluated using the micro-CT technique. For a temperature of 70 °C, the changes occurring in all tested plastics followed a similar pattern. As a result of storing the samples in an oil bath, there was a marked reduction in the internal porosity (Figure 5). This can be observed already after 15 days of storage of the samples. The porosity decreased from the initial value to values close to zero. As the length of the storage time in oil increased, the further decrease in porosity was already insignificant. On this basis, it can be concluded that increased temperature leads to an acceleration of the rate of the porosity change.

**Table 8:** Porosity of the samples stored at a temperature of 23 °C.

Group	ABS	ASA	HIPS	PLA
G0	9.521 (0.78)	2.712 (0.021)	14.831 (1.394)	11.457 (0.982)
G15	0.761 (0.058)	3.741 (0.413)	5.729 (0.623)	1.681 (0.127)
G30	0.883 (0.047)	3.873 (0.293)	1.538 (0.096)	1.175 (0.09)
G60	1.421 (0.167)	4.376 (0.388)	0.651 (0.005)	1.147 (0.163)

(…)—standard deviation.

**Table 9:** Porosity of the samples stored at a temperature of 70 °C.

	ABS	ASA	HIPS	PLA
G0	9.521 (0.871)	2.713 (0.223)	14.835 (0.128)	11.453 (0.098)
G15	0.994 (0.121)	0.962 (0.282)	0.942 (0.112)	0.983 (0.141)
G30	0.992 (0.089)	0.961 (0.083)	0.984 (0.234)	0.988 (0.092)
G60	0.988 (0.113)	0.956 (0.024)	0.993 (0.198)	0.985 (0.105)

(…)—standard deviation.

### 3.4. Statistical Analysis of the Results of Porosity Changes

The existence of statistical differences in the mean porosity values was determined using a one-way ANOVA analysis of variance. The purpose of the analysis was to verify the null hypothesis that the mean values in the study groups are equal in several populations (*k* ≥ 2). In the next step, for the groups between which differences were detected, the Tukey post hoc test was used to assess the significance of the differences. The results of the ANOVA test and the statistical significance of the differences in group mean values are shown in Table 10 and Table 11.

## 4. Discussion

For the ABS specimens, the position of the fatigue curves (Figure 2) also indicates an increase in the fatigue life from the range of the lower *σ_m_* values at both tested temperatures (Table 3). For the higher stress values, above 13–14 MPa, different results were obtained for both temperatures. At 23 °C, the fatigue life decreased in comparison with that of the reference samples, while, at 70 °C degrees, it was close to it. Below this value, the fatigue life increased for all groups stored in oil for both temperatures. For this material, the highest increase in durability was obtained for the samples stored in oil for 60 days.

The results of the cyclic tensile fatigue tests showed, for the ASA material, an increase in the fatigue strength as a result of the mineral oil (Table 4). At room temperature, the number of cycles the material withstood after a storage period in oil of 15 days was the highest. At elevated temperatures, the best results were achieved for the samples that were exposed to mineral oil for 60 days. At this temperature, there was an apparent tendency for the fatigue life to increase as the *σ_m_* stress decreased. The position of the fatigue curves (Figure 3) indicates that the increase in the durability is more significant than the lower value of *σ_m_* than the value close to the strength *σ_m_* of the reference samples. This suggests that increased temperature may increase the penetration of the oil into the structure of the material, and thus the plasticity. This, in turn, increases its cyclic stress strength. With elevated temperature, the effect of the increased strength was even more pronounced. For the third cycle of the test at 50% and 60% of the *σ_m_*, the values reached the limit of the test, which was one million cycles, and the samples did not fail.

For the PLA (Figure 3) specimens at 23 degrees Celsius, no effect of the oil bath and its duration on the change in the fatigue strength was detected, except for the G15 group (Table 5). This suggests that the effect of the oil initially increases, while, in the long term, it decreases the fatigue life. In addition, at 70 °C, changes were observed in the geometry of the specimens, and there was a slight deformation of the shape. This was probably due to the fact that 70 °C is a temperature close to the softening temperature of the material. This suggests that PLA should not be used in working environments where oil is present, especially at elevated temperatures.

For HIPS, the realization of the test at the 80% and 90% *σ_m_* value levels of the reference group was not possible (Figure 5). The samples deteriorated already in the first loading cycle. The probable reason for such a phenomenon was a significant reduction in the *σ_m_* value of the samples stored in oil compared to the *σ_m_* value of the samples in the reference group (Table 6). In all probability, the *σ_m_* value of 90 and 80% of the reference group’s *σ_m_* exceeded that of the samples stored in oil. Tests were only possible at lower load values, suggesting that the HIPS material may not be suitable for applications that require high load values.

The porosity was presented in Figure 6, and Table 8 and Table 9. The temperature of the oil had a generally negative impact on the porosity. At the temperature of 70 °C, the porosity in almost all groups of the materials decreased or was unchanged in comparison to the oil bath at a temperature 23 °C. In the case of ABS G15 and G30, and HIPS G60, the porosity increased with the oil temperature. The oil bath caused the decreased porosity for all materials, except the ASA, where the porosity at G0 was lower than for the other groups. During the investigations, it was not possible to determine the specific impact of the porosity on the fatigue strength. The samples require further chemical analysis to determine the degradation changes. This will be the focus of future research. 

## 5. Conclusions

Mineral oil can act as a kind of “lubrication”, reducing the friction between material particles and filament stitches, and increasing the plasticity of the sample and its cyclic stress toughness. This increase is particularly noticeable and stable for ABS throughout the *σ_m_* stress range at which the tests were conducted. For ASA, it is also visible, but only in the range of Sa stress values below 12–14 MPa, which stands at about 75% of the *σ_m_* value measured for the reference samples made of this material. 

The results obtained for PLA and HIPS indicate that there is no clear relationship between the changes in the cyclic strength and the length of the oil exposure time and temperature. For this reason, based on the results obtained, it is not possible to assess their suitability for such conditions. 

Elevated temperature caused a decrease in the porosity of the samples in most cases. Thus, the homogeneity of the material increased, and the number of geometric notches inside the samples decreased, which also had a beneficial effect on the increase in their cyclic strength.

Based on the results obtained, it can be assumed that the material most suitable for the manufacture of parts operating in the presence of oil and elevated temperatures is ABS. ASA is also suitable, but only in the load range below 75% of the initial *σ_m_* value, as there is a tendency that, the lower the stress, the greater the increase in the cyclic life for ASA. It should be assumed that such high results for ABS were influenced by the reduction in the porosity (much higher than in the case of ASA) and the simultaneous lack of the chemical degradation of the material. The influence of chemical degradation was indicated by the decrease in the fatigue strength for the materials from the G30 and G60 groups compared to the results obtained for the G15 group. However, taking into account the presented research as a whole, it is difficult to determine the cause of this condition. For this purpose, it is necessary to examine and analyze the microstructure of the individual samples. The presented results show that porosity analysis alone is not sufficient in this case. Such research (microstructure analysis) is planned by the authors of this paper for the future.

The above results may have significant implications for industry, particularly in the context of material selection and operation in environments with cyclic stresses and elevated operating temperatures. It should be noted here that other material properties that are relevant to the characteristics of manufactured products, such as hardness, were not studied. In the opinion of the authors, the results obtained can be a starting point for further studies of other mechanical characteristics that are important from the point of view of the requirements for structural elements and machine parts.

## Figures and Tables

**Table 1:** Tensile strength values for the samples from the reference groups.

	ABS	ASA	PLA	HIPS
***σ_m_* (MPa)**	28.5 (0.86)	16.82 (0.31)	29 (0.07)	21.27 (0.37)

(...)—standard deviation. Stress levels in the cyclic tests were set at 90, 80, 70, 60, and 50% in the tensile strength, *σ_m_*, of the material in question.

**Table 2:** Distribution and number of samples used in the micro-CT studies.

	Temperature, 23 °C				Temperature, 70 °C		
	G0	G15	G30	G60	G15	G30	G60
ABS	3	3	3	3	3	3	3
ASA	3	3	3	3	3	3	3
HIPS	3	3	3	3	3	3	3
PLA	3	3	3	3	3	3	3

**Table 3:** Mechanical strength properties under cyclically varying loads for the ABS material.

ABS 23 °C	Preliminary		G15	G30	G60
	Sa, MPa	N, Cycle	N, Cycle	N, Cycle	N, Cycle
90%	25.65	390 ^a^ (14.3)	1819 ^a^ (167.6)	976 ^a^ (68.1)	585 ^a^ (41.5)
80%	22.8	856 ^a^ (43.3)	4454 ^a^ (57.9)	3040 ^a^ (80.7)	3433 ^a^ (64.6)
70%	19.95	2127 ^a^ (97.9)	6751 ^a^ (344.8)	3383 ^a^ (61.6)	3789 ^a^ (63.6)
60%	17.1	6145 ^a^ (149.4)	17,675 ^a^ (257.1)	22,633 ^a^ (485.9)	14,614 ^a^ (309.5)
50%	14.25	16,309 ^a^ (159.9)	168,857 ^a^ (992.5)	89,329 ^a^ (1577.4)	17,738 ^a^ (233.1)
**ABS 70 °C**	**Preliminary**		**G15**	**G30**	**G60**
	**Sa, MPa**	**N, Cycle**	**N, Cycle**	**N, Cycle**	**N, Cycle**
90%	25.65	390 ^a^ (14.3)	494 ^a^ (14)	767 ^a^ (20.4)	756 ^a^ (35.2)
80%	22.8	856 ^a^ (43.3)	2135 ^a^ (47.2)	830 ^a^ (13.1)	4013 ^a^ (41.7)
70%	19.95	2127 ^a^ (98)	3010 ^a^ (77)	4754 ^a^ (54)	2945 ^a^ (43.7)
60%	17.1	6145 ^a^ (149.4)	12,871 ^a^ (112)	9346 ^a^ (122.5)	13,959 ^a^ (217.7)
50%	14.25	16,309 ^a^ (160)	112,641 ^a^ (451.7)	67,562 ^a^ (282.7)	172,590 ^a^ (770)

^a^ Index indicates homogeneous groups within a single material.

**Table 4:** Mechanical strength properties under cyclically varying loads for the ASA material.

ASA 23 °C	Preliminary		G15	G30	G60
	Sa, MPa	N, Cycle	N, Cycle	N, Cycle	N, Cycle
90%	15.14	7628 ^a^ (254.4)	5782 ^a^ (122.1)	3452 ^a^ (57)	10,959 ^a^ (283.1)
80%	13.46	18,190 ^a^ (565.6)	17,079 ^a^ (467.1)	11,581 ^a^ (371.8)	44,485 ^a^ (547.2)
70%	11.77	44,750 ^a^ (544)	81,679 ^a^ (921.6)	65,616 ^a^ (511.1)	117,326 ^a^ (1476.3)
60%	10.09	47,531 ^a^ (427.4)	361,951 ^a^ (1514.5)	216,949 ^a^ (3608)	1,000,000 ^a^ (0)
50%	8.41	99,530 ^a^ (149)	1,000,000 ^a^ (0)	1,000,000 ^a^ (0)	1,000,000 ^a^ (0)
**ASA 70 °C**	**Preliminary**		**G15**	**G30**	**G60**
	**Sa, MPa**	**N, Cycle**	**N, Cycle**	**N, Cycle**	**N, Cycle**
90%	15.14	7628 ^a^ (254.4)	12,944 ^a^ (111.4)	9387 ^a^ (60.5)	21,904 ^a^ (373.1)
80%	13.46	18,190 ^a^ (565.6)	24,858 ^a^ (283)	25,442 ^a^ (445)	85,830 ^a^ (660.4)
70%	11.77	44,750 ^a^ (544)	25,850 ^a^ (777)	108,713 ^a^ (505.1)	324,386 ^a^ (446.2)
60%	10.09	47,531 ^a^ (427.4)	1,000,000 ^a^ (0)	715,529 ^a^ (503.5)	1,000,000 ^a^ (0)
50%	8.41	99,530 ^a^ (149.1)	1,000,000 ^a^ (0)	1,000,000 ^a^ (0)	1,000,000 ^a^ (0)

^a^ Index indicates homogeneous groups within a single material.

**Table 5:** Mechanical strength properties under cyclically varying loads for the PLA material.

PLA 23 °C	Preliminary		G15	G30	G60
	Sa, MPa	N, Cycle	N, Cycle	N, Cycle	N, Cycle
90%	26.1	729 ^a^ (29.1)	922 ^a^ (23.5)	345 ^a^ (16.4)	350 ^a^ (10.4)
80%	23.2	1263 ^a^ (52.3)	1371 ^a^ (77.7)	754 ^a^ (29.1)	731 ^a^ (15.1)
70%	20.3	3280 ^a^ (41.6)	1670 ^a^ (69.5)	1693 ^a^ (31.1)	1372 ^a^ (38.8)
60%	10.095	5038 ^a^ (87.4)	62,637 ^a^ (415.1)	9149 ^a^ (73)	14,169 ^a^ (362)
50%	14.5	39,267 ^a^ (420.6)	12,6834 ^a^ (146.3)	69,085 ^a^ (53.2)	44,676 ^a^ (431)
**PLA 70 °C**	**Preliminary**		**G15**	**G30**	**G60**
	**Sa, MPa**	**N, Cycle**	**N, Cycle**	**N, Cycle**	**N, Cycle**
90%	26.1	729 ^a^ (29.1)	1153 ^a^ (41.6)	144 ^a^ (7.6)	544 ^a^ (20.1)
80%	23.2	1263 ^a^ (52.3)	3129 ^a^ (35.7)	692 ^a^ (10.1)	606 ^a^ (9.4)
70%	20.3	3280 ^a^ (41.6)	3898 ^a^ (32.5)	798 ^a^ (20.6)	1827 ^a^ (39)
60%	17.4	5038 ^a^ (87.4)	7803 ^a^ (38.2)	3851 ^a^ (46.8)	2162 ^a^ (114)
50%	14.5	39,267 ^a^ (420.6)	52,878 ^a^ (146.3)	5130 ^a^ (53.2)	3322 ^a^ (431)

^a^ Index indicates homogeneous groups within a single material.

**Table 6:** Mechanical strength properties under cyclically varying loads for the HIPS material.

HIPS 23 °C	Preliminary		G15	G30	G60
	Sa, MPa	N, Cycle	N, Cycle	N, Cycle	N, Cycle
90%	19.14	-	-	-	-
80%	17.02	-	-	-	-
70%	14.89	-	29 ^a^ (2.8)	4 ^a^ (1.4)	68 ^a^ (4.2)
60%	12.76	43 ^a^ (3.7)	75 ^a^ (5.3)	15 ^a^ (1.2)	384 ^a^ (9.1)
50%	10.64	451 ^a^ (18)	248 ^a^ (8.2)	340 ^a^ (8.6)	748 ^a^ (11.5)
**HIPS 70 °C**	**Preliminary**		**G15**	**G30**	**G60**
	**Sa, MPa**	**N, cycle**	**N, cycle**	**N, cycle**	**N, cycle**
90%	19.15	-	-	-	-
80%	17.02	-	-	-	-
70%	14.89	-	-	12 ^a^ (1.6)	15 ^a^ (2.3)
60%	12.76	43 ^a^ (3.7)	74 ^a^ (4.1)	38 ^a^ (3.3)	49 ^a^ (2.3)
50%	10.64	451 ^a^ (18)	272 ^a^ (6)	125 ^a^ (4.3)	307 ^a^ (5.6)

^a^ Index indicates homogeneous groups within a single material. (-) The inability to study at a given level.

**Table 7:** Equations of the fatigue curves shown in Figure 2, Figure 3, Figure 4 and Figure 5.

	Temp.	ABS	ASA	HIPS	PLA
G0	23 °C	log (N) = −3.01∙log(*σ_a_*) + 43.308	log (N) = −2.61∙log(*σ_a_*) + 38.779	log (N) = −1.062∙log(*σ_a_*) + 16.99	log (N) = −2.861∙log(*σ_a_*) + 43.724
G15	23 °C	log (N) = −2.702∙log(*σ_a_*) + 45.923	log (N) = −1.253∙log(*σ_a_*) + 25.89	log (N) = −1.277∙log(*σ_a_*) + 18.265	log (N) = −1.801∙log(*σ_a_*) + 36.243
	70 °C	log (N) = −2.353∙log(*σ_a_*) + 41.807	log (N) = −1.124∙log(*σ_a_*) + 24.675	log (N) = −1.088∙log(*σ_a_*) + 17.051	log (N) = −3.059∙log(*σ_a_*) + 46.733
G30	23 °C	log (N) = −2.716∙log(*σ_a_*) + 45.94	log (N) = −1.176∙log(*σ_a_*) + 24.64	log (N) = −1.289∙log(*σ_a_*) + 17.454	log (N) = −2.183∙log(*σ_a_*) + 37.752
	70 °C	log (N) = −2.524∙log(*σ_a_*) + 42.771	log (N) = −1.286∙log(*σ_a_*) + 26.747	log (N) = −1.505∙log(*σ_a_*) + 18.195	log (N) = −3.061∙log(*σ_a_*) + 41.721
G60	23 °C	log (N) = −3.584∙log(*σ_a_*) + 51.504	log (N) = −1.312∙log(*σ_a_*) + 27.34	log (N) = −1.004∙log(*σ_a_*) + 18.265	log (N) = −2.17∙log(*σ_a_*) + 37.636
	70 °C	log (N) = −2.241∙log(*σ_a_*) + 41.648	log (N) = −1.553∙log(*σ_a_*) + 30.935	log (N) = −1.308∙log(*σ_a_*) + 18.133	log (N) = −5.455∙log(*σ_a_*) + 59.496

**Table 10:** The significance of the statistical differences between the studied groups for the temperature of 23 °C.

		ABS					ASA		
group	G0	G15	G30	G60	group	G0	G15	G30	G60
G0	-	S	S	S	G0	-	S	S	S
G15	-	-	NS	S	G15	-	-	LD	S
G30	-	-	-	S	G30	-	-	-	S
		**HIPS**					**PLA**		
group	G0	G15	G30	G60	group	G0	G15	G30	G60
G0	-	S	S	S	G0	-	S	S	S
G15	-	-	S	S	G15	-	-	S	S
G30	-	-	-	S	G30	-	-	-	LD

S—statistically significant differences from Tukey’s post hoc test. NS—differences not statistically significant from Fisher’s LSD test. LD—no statistical differences from the ANOVA test.

**Table 11:** The significance of the statistical differences between the study groups for a temperature of 70 °C.

		ABS					ASA		
group	G0	G15	G30	G60	group	G0	G15	G30	G60
G0	-	S	S	S	G0	-	S	S	S
G15	-	-	LD	NS	G15	-	-	NS	LD
G30	-	-	-	LD	G30	-	-	-	LD
		**HIPS**					**PLA**		
group	G0	G15	G30	G60	group	G0	G15	G30	G60
G0	-	S	S	S	G0	-	S	S	S
G15	-	-	S	S	G15	-	-	LD	LD
G30	-	-	-	LD	G30	-	-	-	LD

S—statistically significant differences from Tukey’s post hoc test. NS—differences not statistically significant from Fisher’s LSD test. LD—no statistical differences from the ANOVA test.

## Data Availability

The data supporting this study’s findings are available from the corresponding authors on request. The data are not publicly available due to we have not prepared a data package and it will be used in the next article. They will then be published as a larger data package.

## References

[1] Shahrubudin N., Lee T.C., Ramlan R. (2019). An Overview on 3D Printing Technology: Technological, Materials, and Applications. Procedia Manuf..

[2] Vedrtnam A., Ghabezi P., Gunwant D., Jiang Y., Sam-Daliri O., Harrison N., Goggins J., Finnegan W. (2023). Mechanical Performance of 3D-Printed Continuous Fibre Onyx Composites for Drone Applications: An Experimental and Numerical Analysis. Compos. Part C Open Access.

[3] Vaucher J., Demongeot A., Michaud V., Leterrier Y. (2022). Recycling of Bottle Grade PET: Influence of HDPE Contamination on the Microstructure and Mechanical Performance of 3D Printed Parts. Polymers.

[4] Ranjan R., Kumar D., Kundu M., Chandra Moi S. (2022). A Critical Review on Classification of Materials Used in 3D Printing Process. Mater. Today Proc..

[5] Budzik G., Przeszłowski Ł., Dziubek T., Gontarz M., Dębski M., Smyk E. (2021). Manufacturing Elements with Small Cross-Sections of 17-4 PH Steel (1.4542) with the Application of the DMLS Additive Manufacturing Method. Materials.

[6] Chacón J.M., Caminero M.A., García-Plaza E., Núñez P.J. (2017). Additive Manufacturing of PLA Structures Using Fused Deposition Modelling: Effect of Process Parameters on Mechanical Properties and Their Optimal Selection. Mater. Des..

[7] Letcher T., Waytashek M. (2014). Material Property Testing of 3D-Printed Specimen in PLA on an Entry-Level 3D Printer. Volume 2A: Advanced Manufacturing, Proceedings of the ASME 2014 International Mechanical Engineering Congress and Exposition, Montreal, QC, Canada, 14–20 November 2014.

[8] Kermavnar T., Shannon A., O’Sullivan L.W. (2021). The Application of Additive Manufacturing / 3D Printing in Ergonomic Aspects of Product Design: A Systematic Review. Appl. Ergon..

[9] Jandyal A., Chaturvedi I., Wazir I., Raina A., Ul Haq M.I. (2022). 3D Printing—A Review of Processes, Materials and Applications in Industry 4.0. Sustain. Oper. Comput..

[10] Głowacki M., Mazurkiewicz A., Słomion M., Skórczewska K. (2022). Resistance of 3D-Printed Components, Test Specimens and Products to Work under Environmental Conditions—Review. Materials.

[11] Kumar R., Kumar S. (2020). Trending Applications of 3D Printing: A Study. Asian J. Eng. Appl. Technol..

[12] Espera A.H., Dizon J.R.C., Chen Q., Advincula R.C. (2019). 3D-Printing and Advanced Manufacturing for Electronics. Prog. Addit. Manuf..

[13] Ahmad M.N., Yahya A. (2023). Effects of 3D Printing Parameters on Mechanical Properties of ABS Samples. Designs.

[14] Rawashdeh M.O., Fayyad S.M., Awwad A.S. (2020). Testing Engine Oil Specifications and Properties and Its Effects on the Engines Maintenance and Performance. Wseas Trans. Fluid Mech..

[15] Hozdić E., Hozdić E. (2023). Comparative Analysis of the Influence of Mineral Engine Oil on the Mechanical Parameters of FDM 3D-Printed PLA, PLA+CF, PETG, and PETG+CF Materials. Materials.

[16] Arrêteau M., Fabien A., El Haddaji B., Chateigner D., Sonebi M., Sebaibi N. (2023). Review of Advances in 3D Printing Technology of Cementitious Materials: Key Printing Parameters and Properties Characterization. Buildings.

[17] Sedlak J., Joska Z., Jansky J., Zouhar J., Kolomy S., Slany M., Svasta A., Jirousek J. (2023). Analysis of the Mechanical Properties of 3D-Printed Plastic Samples Subjected to Selected Degradation Effects. Materials.

[18] Müller M., Šleger V., Kolář V., Hromasová M., Piš D., Mishra R.K. (2022). Low-Cycle Fatigue Behavior of 3D-Printed PLA Reinforced with Natural Filler. Polymers.

[19] Ngo T.D., Kashani A., Imbalzano G., Nguyen K.T., Hui D. (2018). Additive manufacturing (3D printing): A review of materials, methods, applications and challenges. Compos. Part B Eng..

[20] Prabhakar M.M., Saravanan A.K., Lenin A.H., Leno I.J., Mayandi K., Ramalingam P.S. (2021). A Short Review on 3D Printing Methods, Process Parameters and Materials. Mater. Today Proc..

[21] Portoacă A.I., Ripeanu R.G., Diniță A., Tănase M. (2023). Optimization of 3D Printing Parameters for Enhanced Surface Quality and Wear Resistance. Polymers.

[22] Senatov F.S., Niaza K.V., Stepashkin A.A., Kaloshkin S.D. (2016). Low-Cycle Fatigue Behavior of 3d-Printed PLA-Based Porous Scaffolds. Compos. B Eng..

[23] Ahmadi R., D’Andrea D., Santonocito D. (2023). Fatigue Assessment of 3D-Printed Porous PLA-Based Scaffold Structures by Thermographic Methods. IOP Conf. Ser. Mater. Sci. Eng..

[24] Svatík J., Lepcio P., Ondreáš F., Zárybnická K., Zbončák M., Menčík P., Jančář J. (2021). PLA Toughening via Bamboo-Inspired 3D Printed Structural Design. Polym. Test..

[25] Rigotti D., Dorigato A., Pegoretti A. (2020). Low-cycle Fatigue Behavior of Flexible 3D Printed Thermoplastic Polyurethane Blends for Thermal Energy Storage/Release Applications. J. Appl. Polym. Sci..

[26] Karim K.I., Mohamad N., Razak J.B.A., Maulod H.E.A., Ahsan Q. (2022). Degradation of Swollen NR/EPDM Filled with Graphene Nanoplatelets in Different Types of Service Oils for Engine Mounting. Int. J. Mater. Prod. Technol..

[27] Topoliński T., Cichański A., Mazurkiewicz A., Nowicki K. (2011). Fatigue Energy Dissipation in Trabecular Bone Samples with Step-wise-Increasing Amplitude Loading. Mater. Test..

[28] Aydemir C., Yenidoğan S., Karademir A., Kandirmaz E.A. (2018). The Examination of Vegetable- and Mineral Oil-Based Inks’ Effects on Print Quality: Green Printing Effects with Different Oils. J. Appl. Biomater. Funct. Mater..

[29] Manufacturer of 3D Printing Filament Spectrumfilaments. 17 March 2024. https://spectrumfilaments.com/en/.

[30] (2012). Plastics—Determination of Mechanical Properties in Static Tension—General Principles.

[31] Głowacki M., Skórczewska K., Lewandowski K., Szewczykowski P., Mazurkiewicz A. (2024). Effect of Shock-Variable Environmental Temperature and Humidity Conditions on 3D-Printed Polymers for Tensile Properties. Polymers.

