# Correction: Głowacki et al. Change in the Low-Cycle Performance on the 3D-Printed Materials ABS, ASA, HIPS, and PLA Exposed to Mineral Oil. *Polymers* 2024, *16*, 1120

**DOI:** 10.3390/polym17030298

**Published:** 2025-01-23

**Authors:** Marcin Głowacki, Adam Mazurkiewicz, Katarzyna Skórczewska, José Miguel Martínez Valle, Emil Smyk

**Affiliations:** 1 Faculty of Mechanical Engineering, Bydgoszcz University of Science and Technology, Kaliskiego 7 Street, 85-796 Bydgoszcz, Poland; adam.mazurkiewicz@pbs.edu.pl (A.M.); emil.smyk@pbs.edu.pl (E.S.); 2 Faculty of Chemical Technology and Engineering, Bydgoszcz University of Science and Technology, Seminaryjna 3 Street, 85-326 Bydgoszcz, Poland; katarzyna.skorczewska@pbs.edu.pl; 3 Department of Mechanics, Building Leonardo Da Vinci, Campus of Rabanales, University of Córdoba, Cta. Madrid-Cádiz, Km. 396, 14071 Córdoba, Spain; jmvalle@uco.es

The authors wish to make a change to their published paper [1]. In the original manuscript, there was a mistake in Table 7 in Section 3.1. The expression “log(*σ_a_*)” was not written in the first part of the equations presented in Table 7. The corrected Table 7 is shown below:

The authors apologize for any inconvenience caused, and the change does not affect the scientific results. This correction was approved by the Academic Editor. The original publication has also been updated.

## Figures and Tables

**Table 7:** Equations of the fatigue curves shown in Figures 2–5.

	Temp.	ABS	ASA	HIPS	PLA
G0	23 °C	log (N) = −3.01∙log(*σ_a_*) + 43.308	log (N) = −2.61∙log(*σ_a_*) + 38.779	log (N) = −1.062∙log(*σ_a_*) + 16.99	log (N) = −2.861∙log(*σ_a_*) + 43.724
G15	23 °C	log (N) = −2.702∙log(*σ_a_*) + 45.923	log (N) = −1.253∙log(*σ_a_*) + 25.89	log (N) = −1.277∙log(*σ_a_*) + 18.265	log (N) = −1.801∙log(*σ_a_*) + 36.243
	70 °C	log (N) = −2.353∙log(*σ_a_*) + 41.807	log (N) = −1.124∙log(*σ_a_*) + 24.675	log (N) = −1.088∙log(*σ_a_*) + 17.051	log (N) = −3.059∙log(*σ_a_*) + 46.733
G30	23 °C	log (N) = −2.716∙log(*σ_a_*) + 45.94	log (N) = −1.176∙log(*σ_a_*) + 24.64	log (N) = −1.289∙log(*σ_a_*) + 17.454	log (N) = −2.183∙log(*σ_a_*) + 37.752
	70 °C	log (N) = −2.524∙log(*σ_a_*) + 42.771	log (N) = −1.286∙log(*σ_a_*) + 26.747	log (N) = −1.505∙log(*σ_a_*) + 18.195	log (N) = −3.061∙log(*σ_a_*) + 41.721
G60	23 °C	log (N) = −3.584∙log(*σ_a_*) + 51.504	log (N) = −1.312∙log(*σ_a_*) + 27.34	log (N) = −1.004∙log(*σ_a_*) + 18.265	log (N) = −2.17∙log(*σ_a_*) + 37.636
	70 °C	log (N) = −2.241∙log(*σ_a_*) + 41.648	log (N) = −1.553∙log(*σ_a_*) + 30.935	log (N) = −1.308∙log(*σ_a_*) + 18.133	log (N) = −5.455∙log(*σ_a_*) + 59.496
